# Developing Valid and Feasible Measures of Sexual Consent for Experience Sampling Methodology

**DOI:** 10.1080/00224499.2021.1907526

**Published:** 2021-04-23

**Authors:** Malachi Willis, Kristen N. Jozkowski, Ana J. Bridges, Robert E. Davis, Jennifer C. Veilleux

**Affiliations:** a Institute of Health and Wellbeing, MRC/CSO Social and Public Health Sciences Unit, University of Glasgow; b Department of Applied Health Science and Kinsey Institute, Indiana University; c Department of Psychological Science, University of Arkansas; d Department of Health, Human Performance, and Recreation, University of Arkansas

## Abstract

Preliminary evidence indicates that people’s sexual consent (i.e., their willingness to engage in sexual activity and communication of that willingness) varies across time and context. Study designs that assess sexual consent at multiple time points (e.g., experience sampling methodology [ESM]) are needed to better understand the within-person variability of sexual consent. However, extant validated measures of sexual consent are not appropriate for ESM studies, which require shorter assessments due to the increased burden this methodology has on participants. As such, the goal of the present study was to develop ESM measures of sexual consent based on items that have previously been validated for use in cross-sectional surveys. We selected items that balanced face validity as evidenced by cognitive interviews (*n* = 10) and content validity as evidenced by experts’ ratings (*n* = 6). To assess the construct validity and feasibility of these items, we administered the selected ESM measures of sexual consent in a seven-day pilot study (*n* = 12). The results suggested that the ESM measures developed in the present study were a valid and feasible assessment of people’s experience-specific internal consent feelings and external consent communication. We conclude with recommendations for sex researchers interested in ESM.

## Introduction

In the academic literature, there are two primary definitions of sexual consent (Hickman & Muehlenhard, 1999; Muehlenhard et al., 2016). First, sexual consent has been conceptualized as an internal feeling of willingness to engage in sexual activity. A second definition indicates that sexual consent constitutes the use of words or behaviors to communicate to another person agreement to engage in sexual activity; signals might be explicit or implicit. Based on these conceptual definitions, measures have been developed and validated to assess the various types of internal consent feelings and external consent communication (e.g., the Internal and External Consent Scales; Jozkowski et al., 2014).

Extant research indicates that sexual consent is complex and contextual – potentially varying from one experience to the next (Willis & Jozkowski, 2019). However, to our knowledge, validated measures of internal and external sexual consent have only been developed for and used in retrospective cross-sectional studies, which are not well-equipped to account for within-person variability (Csikszentmihalyi & Larson, 2014). Validated measures are needed to bolster the credibility of findings regarding the within-person variability of sexual consent. In the present study, we sought to develop valid measures of sexual consent that would be appropriate for experience sampling methodology (ESM).

### Variability of Sexual Consent

Most of the previous studies assessing the nuances of sexual consent have investigated the between-person variability of internal consent feelings and external consent communication. For example, sexual consent can vary by gender (Jozkowski & Peterson, 2013), age (Willis, Blunt-Vinti et al., 2019), or race/ethnicity (Walsh et al., 2019). However, little is known regarding the within-person variability of internal or external sexual consent. Previous studies on how sexual consent varies by context between people have provided initial evidence that a person’s consent can depend on the situation. For example, researchers have consistently shown that sexual consent can vary by type of sexual behavior (Marcantonio et al., 2018; Willis, Hunt et al., 2019), whether alcohol was consumed (Jozkowski & Wiersma, 2015), and being in a private versus a social setting (Jozkowski et al., 2018; Jozkowski & Willis, 2020). While these contextual factors give insight into the potential within-person variability of sexual consent, they have typically been assessed cross-sectionally. As such, most conclusions drawn from previous research on the contextual nuances of sexual consent are based on between-person differences at a single moment in time – rather than within-person differences across time.

Therefore, to assess potential fluctuations due to relevant situational contexts, a few research teams have asked participants about sexual consent multiple times over a study period (e.g., using daily diaries). For example, using open-ended data from a 30-day daily diary study, Willis and Jozkowski (2019) found that whether sexual consent was reportedly communicated varied not only between people but also within people and across events. However, that study and others (O’Sullivan & Allgeier, 1998; Vannier & O’Sullivan, 2011) presented the quantitative data as an aggregate; therefore, the literature still lacks an adequate assessment of how sexual consent might vary from experience to experience. Willis and Jozkowski (2019) urged researchers to employ methodologies and analyses that can estimate the potential variation in sexual consent across contexts.

### Experience Sampling Methodology

One potential approach to investigating how sexual consent varies from experience to experience is ESM, which can be used to ask participants to provide systematic self-reports at multiple points throughout a day (Csikszentmihalyi & Larson, 2014). However, researchers interested in using ESM to examine the within-person variability of sexual consent may lack the tools to do so; existing measures of sexual consent either have been designed for lengthier cross-sectional surveys (e.g., Jozkowski et al., 2014) or have not endured a rigorous validation process (O’Sullivan & Allgeier, 1998; Vannier & O’Sullivan, 2011; Willis & Jozkowski, 2019).

ESM (also referred to as ecological momentary assessment) refers to a range of study designs that can be used to examine within-person variability across experiences. By obtaining multiple data points from participants during a study period, the goal of this methodology is to create a representative sample of people’s experiences (Csikszentmihalyi & Larson, 2014). In this way, ESM provides three notable advantages over traditional retrospective cross-sectional survey designs.

First, by collecting data in the moment (or close to it), ESM studies lessen the need for participants to recollect and reconstruct their memories – processes that are prone to biases (Csikszentmihalyi & Larson, 2014; Iida et al., 2012). Even though people typically feel confident in their memories, evidence indicates that errors made in recalling past experiences reduce the validity and reliability of retrospective self-reported data (Shiffman et al., 2008). Second, by collecting data in everyday settings, ESM studies improve the ecological validity of their findings (Csikszentmihalyi & Larson, 2014; Myin-Germeys et al., 2009). Because participants provide their self-reported data in their typical environment rather than a laboratory setting, responses to ESM surveys more accurately represent natural experiences (Shiffman et al., 2008; Van Berkel et al., 2017). Third, by collecting multiple points of data for each participant, ESM studies can assess within-person variability (Csikszentmihalyi & Larson, 2014; Myin-Germeys et al., 2009). While some ESM studies aggregate these repeated measures to infer a participant’s typical state, researchers more often seek to capitalize on the temporal clarity afforded by repeated measures to assess experience-to-experience variations (Scollon et al., 2009; Shiffman et al., 2008). Overall, collecting momentary self-reported data in people’s natural environments and across multiple time points allows researchers to reduce biases, increase validity, and uncover nuances that might otherwise be masked by cross-sectional correlations.

### Developing and Validating ESM Measures

When developing ESM measures, researchers commonly try to minimize the number of items for each construct (Myin-Germeys et al., 2018; Van Berkel et al., 2017). Using fewer items mitigates some of the burdensome and time-consuming qualities of ESM studies. The use of a few items is not a problem for ESM data because the repeated assessments serve as multiple indicators that reduce random measurement error (e.g., Csikszentmihalyi & Larson, 2014; Schimmack & Grob, 2000; Shiffman et al., 2008). Adding redundant items for the sake of increasing reliability and reducing measurement error may actually decrease rates of compliance (Stone et al., 2003) or reduce the quality of the data (Schimmack, 2003). For these reasons, ESM measures are generally recommended to be as brief as possible.

While some researchers suggest that at least three items be used to measure each ESM construct (Shrout & Lane, 2012), using single items for constructs in ESM studies is widely adopted and typically deemed acceptable (Fisher & To, 2012; Myin-Germeys et al., 2018). Researchers tend to make subjective decisions about which items to include in a truncated scale because few measures have been validated for use in ESM studies (Ebner-Priemer & Trull, 2009). However, for a single item to be considered acceptable for use in an ESM study, it must demonstrate face validity and content validity as well as expected associations with other variables, suggesting construct validity (Fisher & To, 2012).

To design appropriate single-item ESM measures, researchers can first conduct cognitive interviews to assess proposed items for face validity (Myin-Germeys et al., 2018; Shiffman et al., 2008). Cognitive interviews are conducted prior to survey administration with the ultimate goal of better understanding how participants process and respond to items (Willis, 2004). Second, researchers can ensure the content validity of ESM measures by obtaining expert ratings for proposed items (Cheng et al., 2016). From these ratings, researchers can calculate scores that indicate how well items map onto their intended operational definition (e.g., indexes of item-objective congruence; Turner & Carlson, 2003). Third, researchers can provide evidence for the construct validity of ESM measures by conducting pilot tests (Shiffman et al., 2008; Versluis et al., 2018). Further, given the taxing qualities of ESM protocols, piloting measures for this methodology for several days is critical to assess their functionality and feasibility (Fisher & To, 2012).

### Present Study

Rather than writing bespoke items for ESM studies, Ebner-Priemer and Trull (2009) encouraged researchers to develop and use standardized ESM measures – which would facilitate cross-study comparisons. To our knowledge, no ESM measures have been validated to assess sexual consent. Therefore, we sought to design measures that capture how sexual consent can vary from experience to experience. Specifically, we used two phases to develop and validate measures of internal consent feelings and external consent communication intended for use in ESM studies.

The primary goal of Phase 1 was to select items from previous measures of sexual consent that demonstrated both face validity and content validity. Specifically, we sought to identify items used in retrospective cross-sectional research on sexual consent (e.g., Jozkowski et al., 2014; Willis, Blunt-Vinti, et al., 2019) that best represented the constructs of interest. We operationally defined these constructs based on seminal theoretical research on sexual consent (Hickman & Muehlenhard, 1999) and reviews of the academic literature on sexual consent (Muehlenhard et al., 2016). Table 1 presents the operational definitions we used to determine whether the items validly measured constructs related to internal consent feelings (i.e., physical response, safety/comfort, arousal, agreement/want, readiness) and external consent communication (i.e., explicit, implicit, verbal, nonverbal) – from the perspectives of participants (i.e., face validity) and experts (i.e., content validity).

**Table 1.** Operational definitions for each measured aspect of sexual consent

Aspect of Sexual Consent	Operational Definition
Internal Consent Feelings	
Physical Response	Feelings associated with the body’s automatic response to an engaging or exciting stimulus
Safety/Comfort	Feelings associated with a calm assurance that everything will be okay and reflecting the absence of worry or distress
Arousal	Feelings associated with being titillated or drawn to engaging in sexual activity
Agreement/Want	Aspects of a sexual encounter that make it seem to have been a willing and desired interaction between those involved
Readiness	Feelings associated with a confidence that one is prepared to engage in sexual activity
External Consent Communication	
Explicit	Communication that people will most likely understand at face-value – without much subtext or hinting (Explicit cues may be verbal or nonverbal)
Implicit	Communication that people may or may not understand at face-value – but likely involves subtext or hinting (Implicit cues may be verbal or nonverbal)
Verbal	Communication that relies on words; as such, people can say things to express an intention or desire (Verbal cues may be explicit or implicit)
Nonverbal	Communication that does not rely on words; rather, people can do something or move part of their body to express an intention or desire (Nonverbal cues may be explicit or implicit)

The primary goal of Phase 2 was to assess the construct validity of items selected in Phase 1. At the event-level, internal and external sexual consent are related (Willis, Blunt-Vinti, et al., 2019), and there are several other constructs conceptually related to both aspects of consent. Regarding internal consent, researchers have speculated that these feelings are conceptually associated with sexual satisfaction (Marcantonio et al., 2020). Further, women report greater feelings of internal consent during sexual encounters that involved vaginal-penile sex compared with those that involved other sexual behaviors (e.g., genital touching or oral sex; Marcantonio et al., 2018). Regarding external consent, using explicit cues to communicate consent is conceptually associated with initiating sexual activity (Muehlenhard et al., 2016). And for people in committed relationships, consuming alcohol before or during sexual activity is not associated with internal consent feelings or external consent communication (Jozkowski & Wiersma, 2015). Therefore, we assessed whether the ESM measures of sexual consent developed in Phase 1 produced these same associations (i.e., convergent validity) or lack thereof (i.e., discriminant validity) at the event level using data from a seven-day pilot ESM study.

A secondary goal of the pilot study was to assess the functionality and feasibility of the ESM measures of sexual consent. Specifically, we examined person-level descriptive statistics to assess whether these ESM measures could capture within-person variability of internal and external sexual consent. We also asked pilot participants to provide feedback on the items and report their subjective reactions to participating in an ESM study on sexual consent.

## Phase 1: Developing the ESM Measures

### Method

#### Measures

##### Internal Consent Feelings

The 25-item Internal Consent Scale (ICS; Jozkowski et al., 2014) is the only measure of internal consent whose psychometric properties have been publicly validated. The robust measurement properties of the Internal Consent Scale have been replicated in multiple samples (Walsh et al., 2019; Willis, Blunt-Vinti, et al., 2019). The ICS asks participants to indicate how much they experienced a variety of feelings during their most recent partnered sexual activity (Jozkowski et al., 2014). We sought to identify one item to represent each of the five factors of this scale: physical response, safety/comfort, arousal, agreement/want, readiness. Response options were on a four-point Likert-type scale (“Strongly disagree” to “Strongly agree”).

##### External Consent Communication

Jozkowski et al. (2014) also developed an External Consent Scale. However, this measure does not directly map onto Hickman and Muehlenhard’s (1999) conceptualization of external consent communication, which has been used in several recent studies (e.g., Jozkowski et al., 2016; Willis, Blunt-Vinti et al., 2019). Specifically, Jozkowski et al.’s (2014) items may be too specific to fully encompass the myriad ways people communicate consent. As such, in the present study, we adopted the broader classification of external consent, which maintains that people can actively communicate their consent using four different types of general cues: explicit verbal, explicit nonverbal, implicit verbal, and implicit nonverbal (Hickman & Muehlenhard, 1999). Based on this conceptualization of external consent communication (Table 1), we drafted original items that asked participants about how they communicated their willingness to engage in sexual activity during their most recent partnered sexual activity. To write these items, we adopted language and phrases related to consent cues (i.e., explicit/direct, implicit/indirect, verbal, nonverbal) that have been used in previous studies (Hickman & Muehlenhard, 1999; Jozkowski et al., 2016; Willis, Blunt-Vinti, et al., 2019). We then consulted dictionaries and thesauruses to determine other possible phrasings to provide participants; this process resulted in 20 total items. Response options for these items during the cognitive interviews were listed on the same four-point Likert-type scale used by the ICS.

#### Cognitive Interviews

To assess the face validity of items designed to measure internal and external sexual consent, we conducted cognitive interviews with a group of people similar to the intended participants (i.e., sexually active adults). Because the constructs related to internal consent feelings and external consent communication are associated and the items can reflect intricate distinctions, we used concurrent probing, which involves participants’ engagement in certain tasks in a particular order: (1) responding to survey items related to a particular construct, (2) responding to probes related to those items, (3) responding to survey items related to the next construct, (4) responding to probes related to those items, and so on (Willis, 2004). The concurrent approach tends to be preferred to retrospective probing because it allows the researcher to inquire about cognitive processes within moments after they occurred – rather than waiting to do so after the entire survey, which risks the participant forgetting their thought patterns and potentially fabricating their responses to the interviewer’s probes (Willis, 2004).

##### Participants

We conducted 10 cognitive interviews with people who were at least 18 years old. To increase the likelihood that participants would be able to draw from multiple sexual experiences with the same partner (i.e., event-to-event variability), we also required that those participating in the cognitive interviews be in a committed sexual relationship at the time of data collection. A sample size of 10 was determined *a priori* for the present study because this is typically sufficient to reach saturation (Willis, 2004). On average, these participants were 25.0 years old (*SD* = 6.8), ranging from 18 to 39. Regarding gender, five identified as women, four as men, and one as nonbinary. Regarding race/ethnicity, four participants identified as White or European American, four as Asian or Asian American, one as Hispanic or Latin American, and one as Black or African American. Regarding sexual orientation, seven participants identified as heterosexual, two as bisexual, and one as queer. Participants had been with their current sexual partner for an average of 46.8 months (*SD *= 57.8), ranging from 6 to 201.

##### Procedure

We recruited cognitive interview participants via a campus-wide e-newsletter at a university in the southern United States. Participants met the interviewer in a lab setting or in a private study room at the university’s library. They were provided consent forms, which they signed if they were willing to participate. All interviews were recorded on an iPhone using the Voice Recorder application. Each interview was structured as an iterative process in which participants first responded to items on a laptop using Qualtrics survey software for a specific aspect of sexual consent (e.g., consent feelings related to physical response; explicit verbal consent communication). The first part of the interview investigated items measuring each aspect of internal consent feelings; the second part focused on external consent communication. Within each aspect of sexual consent, the items were randomly presented. These items were presented by factor, and the first author asked a structured set of follow-up questions after each factor to determine which items best demonstrated face validity and feasibility within a given aspect of sexual consent (Table 2). Based on interviewer notes and relistening to the audio recordings, the first author synthesized responses by tabulating which items each participant preferred or disliked for each aspect of internal and external sexual consent as well as their rationale for these preferences. This procedure for these cognitive interviews was approved by the university’s institutional review board.

**Table 2.** Structured concurrent cognitive interview prompts

Type of Sexual Consent
Internal Consent Feelings
What did this series of feelings seem to be getting at?
Which of these words best captures [insert previous response]?
Can you tell me why you chose this word?
Are there any other words not listed here that you think would be better?
Do these words reflect being willing to engage in sexual activity?
Were any of these words weird?
Were any of these questions difficult to answer?
Are there any other feelings that you associate with consenting to sexual activity?
External Consent Communication
For these words, how would you define the type of communication being described?
What are examples of signals of sexual consent that are [insert previous response]?
Which of these words best captures [insert previous response]?
Can you tell me why you chose this word?
Are there any other words not listed here that you think would be better?
Were any of these words weird?
Were any of these questions difficult to answer?
Is there a better word for “signal?”

#### Expert Ratings

To assess the content validity of items designed to measure internal and external sexual consent, we obtained ratings from experts regarding how well the items mapped onto their intended operational definitions. Based on these expert ratings, we calculated indexes of item-objective congruence (IIOCs), which are useful for providing an assessment of the content validity of items before pilot testing (Turner & Carlson, 2003). The items included in this IIOC assessment were the same as those from the cognitive interviews.

##### Procedure

We invited three content experts (i.e., scholars who had published peer-reviewed research on sexual consent) and three measurement experts (i.e., researchers who had doctoral training in psychometrics) to rate how well these potential items mapped onto our operational definitions for the various aspects of internal consent feelings and external consent communication (Table 1). Blind to each item’s intended operational definition, the experts rated how well each item measured each objective: 1 (clearly measuring), −1 (clearly not measuring), or 0 (degree that it measures the content area is unclear). Based on the formula and recommended cutoff of .75 provided by Turner and Carlson (2003), we calculated IIOC values to identify items that had higher content validity.

### Results

The empirical data from the cognitive interviews and expert ratings were used to guide decisions regarding which item best represented each operational definition related to internal consent feelings and external consent communication (see Online Supplementary Material for exact IIOC values for each item). Specifically, we balanced face validity and content validity to select items as recommended by Fisher and To (2012). The decision-making process for each construct is described below; there tended to be a clear item that was positively endorsed by both the participants and the experts.

#### Internal Consent Feelings

##### Physical Response

Cognitive interview participants identified these items from the ICS as measuring physical reactions to sexual activity. Participants indicated that “eager” is a more comfortable word than the other options and that it can encompass the other feelings listed in this factor. While several participants thought that “lustful” might best capture the other words and is easy to understand, others were concerned that this word was more abrasive. Even though “erect/vaginally lubricated” was thought to be direct and obvious, participants believed these words might be too scientific or even seen as uncomfortable. Participants consistently disliked “rapid heartbeat” and “flushed” – associating the first with anxiety and the latter with embarrassment.

There was not an obvious item that the experts thought best represented physical response. “I felt eager” and “I felt lustful” were rated as clearly not measuring their intended operational definition. The other three items were in a similar range that was lower than Turner and Carlson’s (2003) recommended cutoff value of .75. Physical response was the most difficult aspect of sexual consent to reconcile across the cognitive interviews and expert ratings. Because “I felt erect/vaginally lubricated” was moderately endorsed by both groups, this item was selected to represent physical response.

##### Safety/Comfort

Participants identified these items from the ICS as measuring feelings of security and being at ease. The item that was consistently liked and not at all disliked was “I felt comfortable.” Participants tended to think that if a person is comfortable then they would experience all of the other feelings (e.g., safety, security). While “safe” and “secure” were also commonly endorsed, participants thought that “comfortable” may be more encompassing. For example, participants more often indicated that a person would likely be safe and secure if they were comfortable; however, they would not necessarily be comfortable if they were safe and secure. Some participants did not like “in control” or “protected” because these words made them think that the sexual activity was not equal or mutual.

The experts rated four items as mapping very well onto their intended operational definition for safety/comfort: “I felt secure,” “I felt protected,” “I felt safe,” and “I felt comfortable.” “I felt in control” was above the .75 cutoff but noticeably lower than the top four, and “I felt respected” was below this cutoff. “I felt certain” was rated as clearly not measuring its intended operational definition. Because “I felt comfortable” was consistently liked by cognitive interview participants and rated as clearly measuring this aspect of consent by the experts, this item was selected to represent safety/comfort.

##### Arousal

Participants identified these items from the ICS as measuring psychological or mental reactions to the prospect of sexual activity. This set of words was often contrasted with the first set, which participants identified as a more physiological arousal. Participants typically liked both “I felt aroused” and “I felt turned on.” The reasons for personal preferences regarding these words were consistent. Participants who preferred “aroused” stated that this term is more physical, sexual, and clinical than “turned on;” according to some participants, these aspects might make it a better choice. Those that preferred “turned on” thought that this phrase meant sexually aroused but that it included more of a mental or emotional quality that “aroused” did not. For this reason, we considered the latter to be the better option given that participants generally identified this set of words as describing more of a psychological experience. Participants consistently disliked “interested” – citing that this word was too innocuous.

The experts rated two items above the .75 cutoff: “I felt aroused” and “I felt turned on.” While the first was rated as a better fit for this construct, its IIOC value was not markedly higher than the second. “I felt interested” was rated as clearly not measuring its intended operational definition. Because cognitive interview participants identified “I felt turned on” as being more psychological than physical (which aligned better with our operational definitions) and experts rated it as clearly measuring this aspect of consent, this item was selected to represent arousal.

##### Agreement/Want

Participants identified these items from the ICS as measuring whether the sexual activity was mutual and everyone involved was okay with it. The item that was consistently liked was “The sexual activity itself felt consensual.” Participants thought that this phrase was clear and seemed the most mutual; they indicated that “consensual” includes both people, whereas the items “consented to” or “agreed to” sounded like they reflected a single person’s perspective. For these reasons, several participants actually disliked “consented to” and “agreed to.” Some also considered these less favorable terms to be too legal.

All five items were above the .75 cutoff for the experts’ ratings. The two highest rated items were “The sexual activity itself felt agreed to” and “The sexual activity itself felt wanted.” Additionally, “The activity itself felt consensual” was in a similarly high range. Because cognitive interview participants thought “The sexual act itself felt consensual” was the most mutual and the experts rated it as clearly measuring this aspect of consent, this item was selected to represent agreement/want.

##### Readiness

Participants identified these items from the ICS as measuring whether people were confident that they wanted to engage in sexual activity. Participants typically liked both “I felt sure” and “I felt willing.” Participants who preferred “sure” thought that this term was the strongest and best encapsulated the others; more often than not, “sure” was seen as more definite and less ambiguous than “willing.” “Ready” was also supported by some participants; however, there were not well-articulated justifications for selecting this term. Finally, some participants did not think that “aware of my surroundings” fit in with the other words because it made them think of being intoxicated or incapacitated. Although this conceptualization was consistent with the original intent during initial development, participants in the cognitive interviews did not find it to be an ideal assessment of readiness.

The experts rated only “I felt ready” above the .75 cutoff. “I felt sure” was well below this cutoff; “I felt willing” and “I felt aware of my surroundings” were both rated as clearly not measuring their intended operational definition. Because “I felt ready” was the only item that experts rated as clearly measuring this aspect of consent and cognitive interview participants endorsed it even though they provided stronger rationales for “I felt sure” and “I felt willing,” this item was selected to represent readiness.

#### External Consent Communication

##### Explicit

Participants identified these items as measuring communication that is easy to interpret. The items that were consistently liked by participants were “I used straightforward signals to communicate my consent” and “I used clear signals to communicate my consent.” Participants said that these terms were easy to understand and were not confusing. Several participants also liked “obvious;” however, almost as many disliked this word. Terms that people did not like included “overt,” “unambiguous,” and “explicit;” these words tended to require too much thought to interpret or were considered too scientific.

The experts rated five items above the .75 cutoff: “explicit signals,” “clear signals,” “unambiguous signals,” “overt signals,” and “straightforward signals.” While “clear signals” was rated as a better fit for this construct, its IIOC value was not markedly higher. One item was slightly below this cutoff: “obvious signals,” and “direct signals” was well below it. Because cognitive interview participants thought “I used straightforward signals to communicate my consent” was easy to interpret and experts rated it as clearly measuring this aspect of consent, this item was selected to represent explicit consent cues.

##### Implicit

Participants identified these items as measuring communication that is not effective and might be perceived as mixed signals. The items that were consistently liked by participants were “I used subtle signals to communicate my consent” and “I used unclear signals to communicate my consent.” Several participants perceived a nuance regarding “subtle” that distinguished it from the other terms. Specifically, participants indicated that “subtle” communication demonstrates intent; in other words, people actively use “subtle” signals to communicate that they are willing. As such, “subtle” seemed to be more in line with consent communication, while the other terms might be more likely to suggest ambivalence or nonconsent. Therefore, even though “unclear” was liked more than it was disliked, participants did not think that this term was as in line with consent as “subtle.” Participants consistently disliked “covert,” “cryptic,” “ambiguous,” and “implicit.” These words were seen as uncommon or unfamiliar, which resulted in participants spending too much time thinking about what they meant.

The experts rated four items above the .75 cutoff: “implicit signals,” “ambiguous signals,” “cryptic signals,” and “covert signals.” One item was slightly below this cutoff: “subtle;” the other two items were well below it: “unclear signals” and “indirect signals.” Even though “I used subtle signals to communicate my consent” was slightly below the recommended IIOC cutoff, we selected this item for implicit consent communication because participants in the cognitive interviews consistently and clearly distinguished this term as better aligning with purposeful communication of a willingness to engage in sexual activity.

##### Verbal

Participants identified these items as measuring the act of communicating verbally. Participants were split regarding whether they preferred “I used verbal signals to communicate my consent” and “I used words to communicate my consent.” Those that liked “verbal signals” thought that it encompassed the other items and was not as restrictive; however, they had reservations regarding the exact wording. When asked how they might rewrite that item, multiple participants endorsed “I communicated my consent verbally.” Participants who liked “words” thought that it was the simplest and best captured the other terms. Participants consistently and strongly disliked “phrases,” and some did not think that “sentences” adequately captured how people communicate their consent verbally.

All four items were rated by the experts as above the .75 cutoff, and they all had the same IIOC value. Because “I communicated my consent verbally” was consistently liked by cognitive interview participants and its parallel wording was rated as clearly measuring this aspect of consent by the experts, this item was selected to represent verbal consent cues.

##### Nonverbal

Participants identified these items as measuring the act of communicating nonverbally. There did not seem to be a consistently preferred item for this set of cues. Participants occasionally disliked “actions,” “behaviors,” and “body language,” but they thought these words were easy to understand and brought to mind specific examples of communication. At the same time, some participants thought that these terms were too restrictive; for example, it was noted that these terms might not include facial expressions – which were identified as an important aspect of consent communication. As such, “nonverbal signals” was preferred as being the most encompassing. Again, participants noted they would like this item more if it read, “I communicated my consent nonverbally.” Participants did not like “gesture,” thinking it was an odd word and too ambiguous.

The experts rated three items above the .75 cutoff; “nonverbal signals” was rated the highest, and the other two items were closer to the cutoff: “gestures” and “body language.” One item was slightly below this cutoff: “actions,” and “behaviors” was well below it. Because “I communicated my consent nonverbally” was consistently liked by cognitive interview participants and its parallel wording was rated as clearly measuring this aspect of consent by the experts, this item was selected to represent nonverbal consent cues.

## Phase 2: Piloting the ESM Measures

In Phase 2, we piloted the items selected for their face validity and content validity as evidenced by the cognitive interviews and expert rating detailed in Phase 1.

### Method

#### Participants

We piloted the ESM measures of sexual consent with 12 people, which is similar to samples sizes of previous ESM pilot studies (e.g., Cordier et al., 2016 [*n* = 6]; Hare et al., 2016 [*n* = 9]). On average, these participants were 32.5 years old (*SD* = 11.1), ranging from 21 to 58. Regarding gender, eight identified as women and four as men. Regarding race/ethnicity, nine participants identified as White or European American, one as Asian or Asian American, one as Hispanic or Latin American, and one as Black or African American. Regarding sexual orientation, eight participants identified as heterosexual, three as bisexual, and one as pansexual. One participant who identified as a woman reported that their current committed sexual partner was a woman; otherwise, all other participants reported being in mixed-gender relationships. Participants had been with their current sexual partner for an average of 67.5 months (*SD *= 75.1), ranging from 3 to 231.

#### Procedure

We recruited pilot participants via a campus-wide e-newsletter and social media to complete an eligibility screener. Interested people who clicked on the recruitment link were directed to an introductory page that provided them with information about the study and screener questions using Qualtrics survey software. To be eligible, participants had to be at least 18 years old, have daily access to a device supported by iOS (e.g., iPhone) or Android (e.g., smartphone), and be sexually active. Similar to Willis and Jozkowski (2019), we defined “sexually active” as having participated in sexual activity (e.g., passionate kissing, oral sex, vaginal sex, anal sex) on at least two days in the preceding week.

Those eligible were provided a link to the baseline survey that was to be completed via Qualtrics survey software. Participants filled out a baseline survey that included sociodemographic items on a personal computer at a location of their choosing. After reviewing the informed consent form online, participants who wished to participate in the study clicked to the next page to begin the online survey. Those who completed the baseline survey received instructions for downloading the LifeData application^1^ ^1^The LifeData application can prompt participants to complete the ESM surveys, time stamp the responses, and store the data. Due to potential sensitivity of the questions asked in the ESM surveys, it was important to select an application that keeps anonymous records and allows the participant to prevent their data from being used if they wish. The LifeData application does not record any identifying information from the participant’s smartphone and permits participants to delete their data at any time during the study. (lifedatacorp.com) onto their device.

The ESM survey was sent to participants four times a day using a semi-random sampling scheme (i.e., random sampling within four fixed windows every day). The specific windows were 9am–12pm, 12pm–3pm, 3pm–6pm, and 6pm–9pm. If participants engaged in partnered sexual activity since their most recent survey, they filled out the ESM measures of sexual consent as well as other items regarding the sexual encounter. If not, they filled out other items related to their relationship, which was done to make the survey length approximately equal on both tracks – eliminating incentive to receive a shorter ESM survey by falsely reporting a lack of partnered sexual activity (Willis & Jozkowski, 2019).

Finally, at the end of the seven-day ESM study period, pilot participants were invited to complete an exit survey to provide their feedback on the ESM measures of sexual consent. Specifically, we asked participants to “Please indicate whether you thought any of [the statements you responded to in the daily surveys over the past week] did not make sense to you or sounded awkward.” In the exit survey, we also assessed the feasibility of assessing sexual consent using ESM measures by asking whether participating in this study was easy, confusing, interesting, frustrating, fun, and boring (on a five-point Likert scale from “strongly agree” to “strongly disagree”).

Based on the number of ESM surveys they completed, participants received up to a 20 USD Amazon.com e-gift card for their participation. The procedure for this pilot study was approved by the university’s institutional review board.

#### Measures

##### Sexual Behavior

Participants responded to the following prompt: “Since the last beep, I engaged in the following behaviors with my partner.” Response options included passionate kissing, genital touching, oral sex, vaginal sex, and anal sex; participants were instructed to select all that applied. For the purposes of this study, responses were dichotomized: 0 = sexual encounters without vaginal sex and 1 = sexual encounters with vaginal sex.

##### Sexual Initiation

At time points for which participants reported engaging in partnered sexual activity, they were asked “Who initiated this sexual encounter?” Response options included “I did,” “My partner did,” “We both did,” and “I’m not sure.” For the purposes of this study, responses were dichotomized: 0 = sexual encounters the participant did not initiate (i.e., “My partner did” and “I’m not sure”) and 1 = sexual encounters the participant initiated or co-initiated (i.e., “I did” and “We both did”).

##### Sexual Consent

We measured both internal and external sexual consent with the nine items selected in Phase 1. Participants only responded to these items if they reported partnered sexual activity. Items assessing internal consent feelings included “During these sexual behaviors, I felt erect/vaginally lubricated,” “During these sexual behaviors, I felt comfortable,” “During these sexual behaviors, I felt turned on,” “During these sexual behaviors, the sexual act itself felt consensual,” and “During these sexual behaviors, I felt ready.” Items assessing external consent communication included “I used straightforward signals to communicate my willingness to engage in these sexual behaviors,” “I used subtle signals to communicate my willingness to engage in these sexual behaviors,” “I verbally communicated my willingness to engage in these sexual behaviors,” and “I nonverbally communicated my willingness to engage in these sexual behaviors.” Per recommendations on selecting a response format for ESM measures (Fisher & To, 2012; Schimmack, 2003), response options for each of these items measuring sexual consent were provided on aunipolar 11-point sliding scale (“Not at all” to “Very much”).

##### Sexual Satisfaction

If participants reported recent partnered sexual activity, they responded to “I felt satisfied with these sexual behaviors.” Response options were provided on aunipolar 101-point sliding scale (“Not at all” to “Very much”).

##### Alcohol Consumption

If participants reported recent partnered sexual activity, they were asked “About how many alcoholic beverages did you have before engaging in these sexual behaviors?” Response options were provided on a 7-point sliding scale (“0” to “6+”). For the purposes of this study, responses were dichotomized: 0 = sexual encounters that did not involve alcohol consumption and 1 = alcohol-involved sexual encounters.

#### Analysis

We calculated descriptive statistics and bivariate correlations using SPSS 26. Effect sizes for correlations were considered small at .1, medium at .3, and large at .5 (Cohen, 1992). Correlations were tested at an α-level of .05. We examined associations both at the person level and event level. For person-level associations, we calculated mean scores across participants’ responses during the study period. Event-level associations were assessed using all time points as cases; thus, we were unable to account for within-person variability. We also assessed the reliability of the ESM measures of internal and external sexual consent. A Cronbach’s α of 0.70 or greater is widely considered to be an adequate indicator of internal consistency (Taber, 2018).

### Results

#### Person-Level Descriptive Statistics

Of the 336 ESM surveys administered, pilot participants completed 251 (74.7%). Ten of the 12 participants reported at least one instance of sexual behavior during the seven-day ESM study period. For these 10 participants, the average number of sexual encounters was 3.1 (SD = 2.2), ranging from 1 to 7.

The ESM measures successfully captured within-person variability in sexual consent from experience to experience. Pilot participants varied in their reports of internal consent feelings and external consent communication; person-level descriptive statistics for the five ESM items related to internal consent are presented in Table 3, and those for the four ESM items related to external consent are presented in Table 4.

**Table 3.** Pilot study results for ESM measures of internal consent feelings

	Time Points		Physical Response		Safety/Comfort		Arousal		Agreement/Want		Readiness	
	Completed	With Sex. Act.	*M*	*SD*	*M*	*SD*	*M*	*SD*	*M*	*SD*	*M*	*SD*
Participant 1	19	7 (36.8%)	6.7	1.7	8.3	1.1	7.6	0.8	8.5	1.0	8.1	1.1
Participant 2	27	6 (22.2)	9.2	1.3	9.8	0.4	10.0	0.0	9.7	0.8	10.0	0.0
Participant 3	19	5 (26.3)	5.8	3.3	10.0	0.0	8.8	1.6	10.0	0.0	10.0	0.0
Participant 4	11	3 (27.7)	5.0	1.7	10.0	0.0	10.0	0.0	10.0	0.0	10.0	0.0
Participant 5	21	3 (14.3)	10.0	0.0	10.0	0.0	10.0	0.0	10.0	0.0	10.0	0.0
Participant 6	19	2 (10.5)	7.5	0.7	9.0	0.0	8.0	0.0	10.0	0.0	9.0	0.0
Participant 7	28	2 (7.1)	6.5	2.1	9.5	0.7	7.0	1.4	9.5	0.7	9.5	0.7
Participant 8	10	1 (10.0)	9.0	–	10.0	–	8.0	–	10.0	–	10.0	–
Participant 9	14	1 (7.1)	8.0	–	10.0	–	10.0	–	10.0	–	10.0	–
Participant 10	27	1 (3.7)	8.0	–	7.0	–	9.0	–	10.0	–	8.0	–

“With Sex. Act.” refers to the number of time points during the 7-day pilot study that a participant reported engaging in sexual activity with their partner. The value in parentheses is the percentage of completed surveys for which a participant reported partnered sexual activity.

**Table 4.** Pilot study results for ESM measures of external consent communication

	Time Points		Explicit		Implicit		Verbal		Nonverbal	
	Completed	With Sex. Act.	*M*	*SD*	*M*	*SD*	*M*	*SD*	*M*	*SD*
Participant 1	19	7 (36.8%)	5.0	3.5	5.4	3.3	5.1	3.5	8.1	1.1
Participant 2	27	6 (22.2)	7.0	3.0	9.3	1.0	7.0	3.7	9.0	0.9
Participant 3	19	5 (26.3)	10.0	9.6	8.0	0.9	9.4	1.3	9.6	0.9
Participant 4	11	3 (27.7)	8.0	1.7	8.3	1.5	3.7	0.6	10.0	0.0
Participant 5	21	3 (14.3)	10.0	0.0	10.0	0.0	10.0	0.0	10.0	0.0
Participant 6	19	2 (10.5)	7.0	1.4	6.0	2.8	3.0	4.2	9.0	1.4
Participant 7	28	2 (7.1)	9.0	1.4	8.0	0.0	7.0	1.4	8.0	0.0
Participant 8	10	1 (10.0)	8.0	–	8.0	–	8.0	–	8.0	–
Participant 9	14	1 (7.1)	10.0	–	0.0	–	10.0	–	0.0	–
Participant 10	27	1 (3.7)	10.0	–	3.0	–	10.0	–	3.0	–

“With Sex. Act.” refers to the number of time points during the 7-day pilot study that a participant reported engaging in sexual activity with their partner. The value in parentheses is the percentage of completed surveys for which a participant reported partnered sexual activity.

Regarding within-person variability, we found that each pilot participant who reported multiple partnered sexual events during the study period oscillated in their internal consent feelings and external consent communication. Demonstrating this variation in sexual consent across time, two figures in the Online Supplementary Material depict how the three pilot participants with at least five data points of partnered sexual activity varied in their internal and external sexual consent depending on the sexual encounter.

#### Event-Level Associations

At the event level, the five items measuring internal consent feelings were internally consistent (α = .71), as were the four items measuring external consent communication (α = .70). Mean scores for internal and external consent were significantly associated, *r* = .54, *p* = .002; sexual encounters with greater use of consent communication cues had greater levels of consent feelings.

Associations between internal and external sexual consent are presented at the item level in Table 5. Feelings of safety/comfort and readiness were significantly and positively correlated with each type of consent communication, *r*s ≥ .36, *p*s < .050. Feelings of arousal and agreement/want were significantly and positively correlated with some types of consent communication, while feelings of physical response were not correlated with any type (see Table 5).

**Table 5.** Bivariate correlations between internal consent feelings and external consent communication

	IC_P	IC_S	IC_A	IC_W	IC_R	EC_E	EC_I	EC_V
IC_P	–							
IC_S	.15	–						
IC_A	.53**	.40*	–					
IC_W	.14	.77***	.34	–				
IC_R	.17	.95***	.49**	.77***	–			
EC_E	.05	.46**	.27	.53**	.57***	–		
EC_I	.15	.63**	.32	.41*	.62***	.47**	–	
EC_V	.20	.38*	.19	.43*	.46**	.69***	.31	–
EC_N	.03	.43*	.13	.20	.36*	.02	.73***	−.03

Internal consent feelings: physical response (IC_P), safety/comfort (IC_S), arousal (IC_A), agreement/want (IC_W), and readiness (IC_R). External consent communication: explicit cues (EC_E), implicit cues (EC_I), verbal cues (EC_V), nonverbal cues (EC_N).

*p < .05. **p < .01. ***p < .001.

Internal consent feelings were strongly associated with sexual satisfaction, *r* = .67, *p* < .001. While internal consent was not significantly greater for sexual encounters with vaginal sex, there was a medium effect size between these two variables, *r* = .31, *p* = .090. Internal consent feelings were not associated with alcohol consumption at the event-level, *r* = .10, *p* = .598.

External consent communication was significantly and positively associated with sexual initiation, *r* = .40, *p* = .026. Specifically, during sexual encounters that participants were involved in the initiation of, they reported greater use of explicit cues, *r*= .52, *p* = .003, and implicit cues, *r*= .44, *p* = .013; however, sexual initiation was not associated with using either verbal or nonverbal cues. Further, external consent communication had little or no association with alcohol consumption at the event-level, *r* = −.14, *p* = .462.

#### Open-Ended Feedback

When asked which ESM items lacked clarity or sounded awkward, none of the participants selected the items measuring internal consent feelings. However, four of the participants provided critical feedback regarding the ESM measures of external consent communication. Each of these four participants indicated or implied that definitions would have been helpful.

The straightforward and subtle signals terms are never defined. What is considered straightforward/subtle? (31-year-old heterosexual woman)

It is unclear to me what this question (on straightforward signals) means. I’m not sure how you could improve it, except maybe including definitions in the initial survey. (22-year-old bisexual woman)

I believe this question (on subtle signals) is unclear when compared to the nonverbal communication of sexual willingness. What differentiates the two? (27-year-old heterosexual woman)

Define straightforward/give example. Define subtle/give example (it felt similar to ‘nonverbal’). Again, how is nonverbal different than ‘subtle’ communication. (25-year-old bisexual woman)

The other six participants who reported at least one sexual encounter indicated that all the ESM measures of external consent communication made sense and none of them sounded awkward.

#### Closed-Ended Ratings of Feasibility

Indicating the feasibility of assessing experience-to-experience variability in sexual consent using these measures, all 12 pilot participants agreed or strongly agreed that participating in this ESM study was “easy.” Further, all 12 disagreed or strongly disagreed that participating was “confusing.” Only two participants rated this study as “frustrating” or “boring,” while 10 rated it as “fun” and nine as “interesting.”

## Discussion

In the present study, we selected items based on existing cross-sectional measures of sexual consent to be used in ESM studies. We provided evidence for their face validity using cognitive interviews and content validity using experts’ ratings. Further, we conducted a pilot ESM study to demonstrate their construct validity. Using event-level data, we corroborated previous research on sexual consent regarding expected associations or lack thereof. The moderate associations we found between internal and external sexual consent are consistent with previous research and suggest that these types of consent are separate and uniquely contribute to an overall conceptualization of sexual consent (Jozkowski et al., 2014; Walsh et al., 2019; Willis et al., 2021). Further demonstrating convergent validity, we supported theories that internal consent feelings are related to sexual satisfaction (Marcantonio et al., 2020) and that external consent communication is associated with sexual initiation (Muehlenhard et al., 2016). We also supported previous research that engaging in vaginal sex is associated with greater internal consent feelings compared with other sexual behaviors (Marcantonio et al., 2018). Evidencing discriminant validity, we corroborated findings that neither internal nor external sexual consent are associated with alcohol consumption at the event level for people in committed relationships (Jozkowski & Wiersma, 2015). Overall, the results suggested that the ESM measures developed in the present study are a valid and feasible assessment of people’s sexual consent.

### Methodological Considerations

Based on findings from the present study, there are two qualities of the ESM measures we developed that warrant further consideration. First, one of the items selected to assess feelings associated with internal sexual consent did not function optimally. In the pilot study, the item measuring physical response (i.e., “During these sexual behaviors, I felt erect/vaginally lubricated”) was not as strongly correlated with the items measuring the other aspects of internal consent as the intercorrelations of those other items. Selecting an item for the physical response aspect of internal sexual consent that demonstrated face validity and content validity was difficult – participants and experts preferred different items for this construct. It may be that there was not a single item in the Internal Consent Scale that ideally represented physical response – especially when conceptualizing sexual activity more broadly than vaginal-penile sex, which was the behavior of interest when the items for this measure were written (Jozkowski et al., 2014). Indeed, feeling vaginally lubricated or erect may be less reflective of willingness to engage in behaviors like passionate kissing. Researchers should consider how ESM studies might better measure physical response as a potential indicator of willingness for wider ranges of sexual behaviors. That said, the ESM measures of internal sexual consent developed in the present study still demonstrated adequate internal consistency and may be used to validly and reliably assess people’s consent feelings from experience to experience. Researchers using this internal consent measure should simply exercise caution when interpreting this item at time points when participants engaged in partnered sexual activity that did not involve genital stimulation.

Regarding external sexual consent, feedback provided by pilot participants in their open-ended responses indicated that they struggled to distinguish the various constructs of consent communication. Corroborating this feedback, the strong correlations between verbal and explicit cues as well as between nonverbal and implicit cues suggest that participants may have conflated these types of consent communication in their event-level data – even though they are conceptually distinct categorization systems (Hickman & Muehlenhard, 1999; Jozkowski et al., 2016). To help clarify these distinctions in future studies, we recommend that researchers using the ESM measure of external sexual consent provide the operational definitions of straightforward, subtle, verbal, and nonverbal signals (Table 1) to participants at the beginning of the study. Doing so should increase the validity of these items measuring consent communication.

### Implications for Sexual Consent Research

ESM studies on sexual consent may provide further empirical support for the conceptualization that sexual consent is contextual (Willis & Jozkowski, 2019). The existing literature has typically only assessed sexual consent cross-sectionally or used analyses that limit conclusions about within-person variability. However, the ESM measures developed in the present study provide a tool that can clarify the extent that researchers should be considering experience-specific nuances of sexual consent. For example, do internal feelings or external communication of consent from previous sexual encounters affect how consent is experienced during future encounters? Do variations in sexual consent from experience to experience predict constructs like relationship satisfaction or sexual satisfaction? Beginning to answer these types of questions will continue to expand the growing literature on the complexities of sexual consent.

The prospective novel contributions of ESM studies designed to investigate sexual consent have the potential to provide previously unexplored facets of consent for several stakeholders to consider. Researchers might examine how previously supported group differences (e.g., gender or relationship status) might vary based on the context of a sexual encounter. Educators could include the effects of context on sexual consent in their curricula, providing students with a model of consent that might be more applicable to the variability they may experience than a one-size-fits-all approach (e.g., affirmative consent initiatives). Relationship therapists may draw on how circumstances between partners have the ability to influence sexual consent in an attempt to improve communication and relationship satisfaction. Administering the measures developed in this study in ESM research can help realize these implications related to experience-specific correlates of sexual consent.

### Implications for Sex Research

We urge sex researchers interested in using ESM, or similar methodologies, to thoughtfully consider the measures they decide to use. ESM measures should demonstrate face validity, content validity, and construct validity (Fisher & To, 2012), as well as feasibility (Myin-Germeys et al., 2018). However, previous studies investigating people’s daily sexual experiences have not typically provided empirical evidence supporting the validity of their measures. Rather, some sex researchers have adapted items from scales validated for traditional retrospective cross-sectional surveys and presumed their acceptability for ESM studies (e.g., Holland et al., 2017; Shrier & Blood, 2016). Others have administered full scales validated for other methodologies (e.g., Muise et al., 2014; Pâquet et al., 2018), which may be feasible once a day but could become unduly burdensome in study designs that ask participants to respond more frequently. Still other sex researchers (e.g., Kashdan et al., 2018; Willis & Jozkowski, 2019) have seemingly written items that appear to be face valid and content valid but lacked data supporting such assumptions – even if they provided a theoretical rationale for their items (e.g., Fortenberry & Hensel, 2011)

Sex researchers interested in using ESM should emphasize the development of measures that validly assess their constructs of interest and that are feasible for these study designs; doing so will be critical to fully realizing the benefits of ESM: namely, reducing recall bias, increasing ecological validity, and assessing within-person variability. More methodological research that provides robust evidence regarding ESM measures of key constructs in sex research will assist in the standardization and replicability of findings, which could support larger networks of sex researchers using ESM and ultimately generate findings that are reliable and generalizable (Ebner-Priemer & Trull, 2009; Myin-Germeys et al., 2018).

### Limitations and Future Directions

Two noteworthy limitations of the present study and potential areas of future research are that our findings exist within the contexts of committed sexual relationships and events in which partnered sexual activity ultimately occurred. First, additional work is needed to determine whether the measures developed in this study adequately capture within-person variability for people engaging in sexual activity with casual partners because sexual consent varies across relationship types. For example, ratings of internal sexual consent tend to be higher for those in committed relationships versus casual ones (Marcantonio et al., 2018), and people think committed partners do not need to actively communicate their willingness to engage in sexual activity (Humphreys, 2007). Second, we only developed and validated measures of sexual consent within the context of sexual activity that ultimately occurred. However, examining how people experience and communicate sexual ambivalence, unwillingness, or willingness at time points during which no partnered sexual activity occurred would provide further insight regarding the complex nuances that underlie event-specific decisions to engage in partnered sexual activity. As such, ESM measures would also be worth developing for sexual refusals, which previous research indicates exist on similar continua as sexual consent communication (Marcantonio & Jozkowski, 2020).

Further, the sample sizes in the present study were adequate given their intended purposes (i.e., providing preliminary evidence that these measures demonstrate face validity, content validity, and construct validity). However, no generalizable conclusions can be made from these data, and this pilot sample should not be used to assess associations between internal and external sexual consent while accounting for within-person variability. We recommend that future ESM studies investigating sexual consent build on our preliminary evaluation of these measures by collecting data from samples large enough to support their conclusions and to capitalize on this methodology’s ability to assess within-person variability.

While ESM reduces recall bias, other biases may still be relevant. For example, like other study designs, our sampling may have been subjected to a self-selection bias. Since we advertised this study as being on “sexual experiences,” our participants might represent people who are more open and willing to discuss their sex lives. Further, social desirability could have influenced self-reports in this study because we asked about behaviors that people might be inclined to misreport (e.g., sex, alcohol use).

Finally, this study sought to develop valid ESM measures of internal and external sexual consent; yet, a third conceptualization remains: sexual consent perceptions (Muehlenhard et al., 2016). In addition to experiencing feelings associated with a willingness to engage in sexual activity and communicating that willingness to somebody else, people must be able to interpret contextual cues or the communication cues of others that might indicate a person’s willingness to engage in sexual activity. For a more comprehensive assessment of sexual consent using ESM, valid measures should also be developed for consent perceptions. Further, ESM studies that collect data from sexual dyads on these three components of sexual consent would help researchers understand how effective sexual partners are at communicating sexual consent.

### Conclusion

In sum, this study provided valid tools to measure sexual consent in daily life using adapted versions of previous cross-sectional measures. Preliminary data from our pilot study suggested that sexual consent varies over time – consistent with previous work on sexual consent (Willis & Jozkowski, 2019). When using the measures developed in this study, researchers should increase the sample size, increase the study duration, and systematically study variations in internal and external sexual consent while accounting for within-person variability. Overall, this study provided initial evidence that sexual consent can be validly assessed in real life contexts and that ESM studies can enrich our understanding of how contextual sexual consent is.

## Supplementary Material

Supplemental Material
