# Supplementary material for: Developing Valid and Feasible Measures of Sexual Consent for Experience Sampling Methodology
Source: J Sex Res. 2021 Apr 23;58(8):996–1007. doi: 10.1080/00224499.2021.1907526 (PMC9239692; doi:10.1080/00224499.2021.1907526)

Online Supplementary Material

Supplemental Table 1

*Index of Item-Objective Congruence Values for the Items Measuring Internal Consent Feelings*

| Factor | Item Wording | IIOC Value |
| --- | --- | --- |
| Physical Response | I felt rapid heartbeat. | .685 |
|  | I felt flushed. | .667 |
|  | I felt eager. | -.037 |
|  | I felt lustful. | -.056 |
|  | I felt erect/vaginally lubricated. | .593 |
| Safety/Comfort | I felt secure. | .971 |
|  | I felt protected. | .963 |
|  | I felt safe. | .963 |
|  | I felt respected. | .704 |
|  | I felt certain. | -.296 |
|  | I felt comfortable. | .944 |
|  | I felt in control. | .852 |
| Arousal | I felt aroused. | .833 |
|  | I felt turned on. | .778 |
|  | I felt interested. | .222 |
| Agreement/Want | The sexual activity itself felt consented to. | .815 |
|  | The sexual activity itself felt agreed to. | .889 |
|  | The sexual activity itself felt wanted. | .889 |
|  | The sexual activity itself felt consensual. | .852 |
|  | The sexual activity itself felt desired. | .796 |
| Readiness | I felt ready. | .870 |
|  | I felt sure. | .519 |
|  | I felt willing. | -.185 |
|  | I felt aware of my surroundings. | -.148 |

*Note.* The recommended cut-off value for an item that measures its intended operational definition well is .75 (Turner & Carlson, 2003).

Supplemental Table 2

*Index of Item-Objective Congruence Values for the Items Measuring Internal Consent Feelings*

| Factor | Item Wording | IIOC Value |
| --- | --- | --- |
| Physical Response | I felt rapid heartbeat. | .685 |
|  | I felt flushed. | .667 |
|  | I felt eager. | -.037 |
|  | I felt lustful. | -.056 |
|  | I felt erect/vaginally lubricated. | .593 |
| Safety/Comfort | I felt secure. | .971 |
|  | I felt protected. | .963 |
|  | I felt safe. | .963 |
|  | I felt respected. | .704 |
|  | I felt certain. | -.296 |
|  | I felt comfortable. | .944 |
|  | I felt in control. | .852 |
| Arousal | I felt aroused. | .833 |
|  | I felt turned on. | .778 |
|  | I felt interested. | .222 |
| Agreement/Want | The sexual activity itself felt consented to. | .815 |
|  | The sexual activity itself felt agreed to. | .889 |
|  | The sexual activity itself felt wanted. | .889 |
|  | The sexual activity itself felt consensual. | .852 |
|  | The sexual activity itself felt desired. | .796 |
| Readiness | I felt ready. | .870 |
|  | I felt sure. | .519 |
|  | I felt willing. | -.185 |
|  | I felt aware of my surroundings. | -.148 |

*Note.* The recommended cut-off value for an item that measures its intended operational definition well is .75 (Turner & Carlson, 2003).

*Supplemental Figure 1.* Internal consent feelings over the seven-day study period for three example participants to demonstrate the day-to-day within-person variability of this construct.


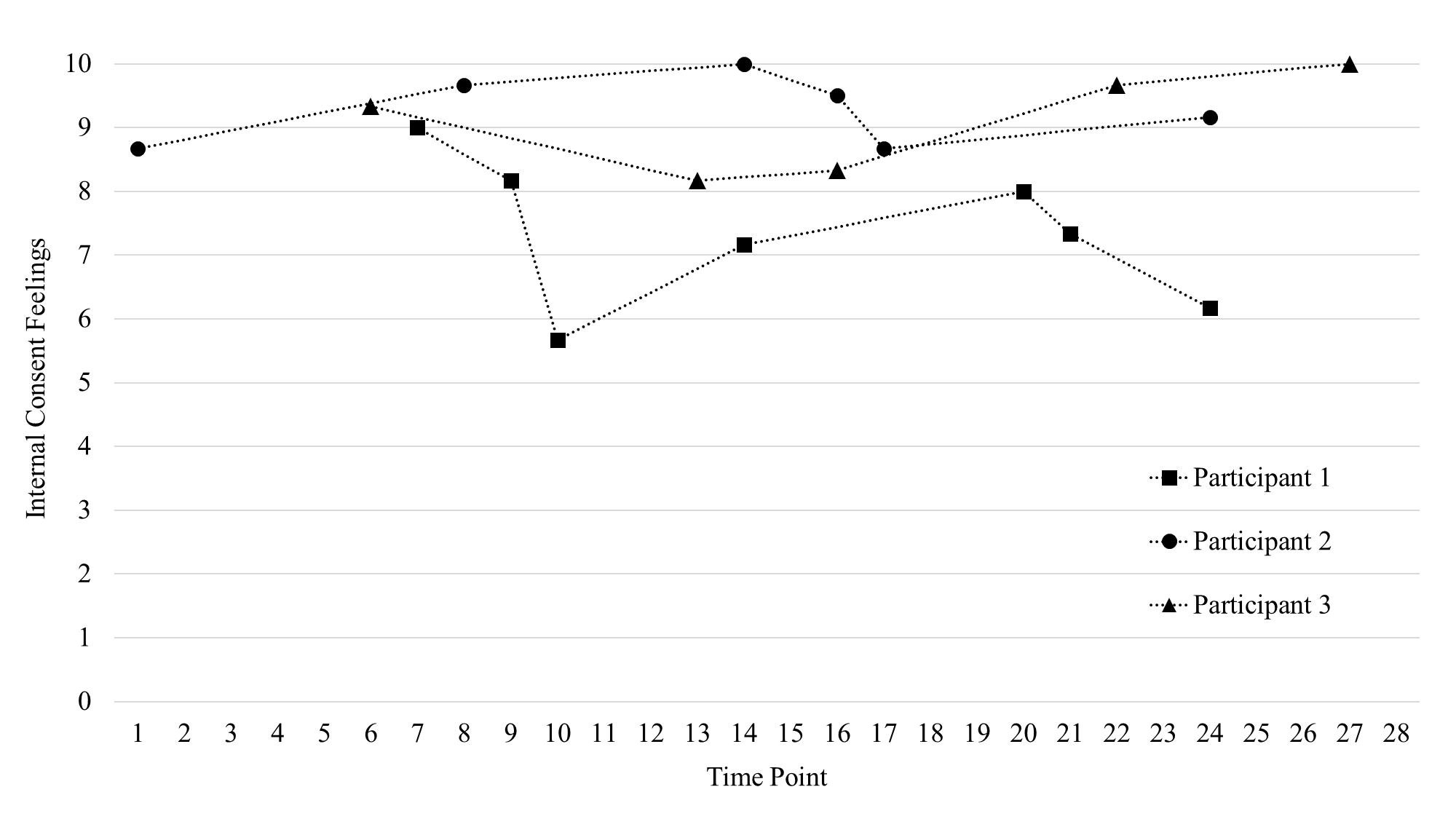


*Supplemental Figure 2.* External consent communication over the seven-day study period for three example participants to demonstrate the day-to-day within-person variability of this construct. Based on our operational definitions, we conceptualized external consent communication as comprising two independent continua. The explicit-implicit continuum (x-axis) was plotted by averaging the score for the “straightforward” item with the reversed score for the “subtle” item. The verbal-nonverbal continuum (y-axis) was plotted by averaging the score for the “verbally” item with the reversed score for the “nonverbally” item.


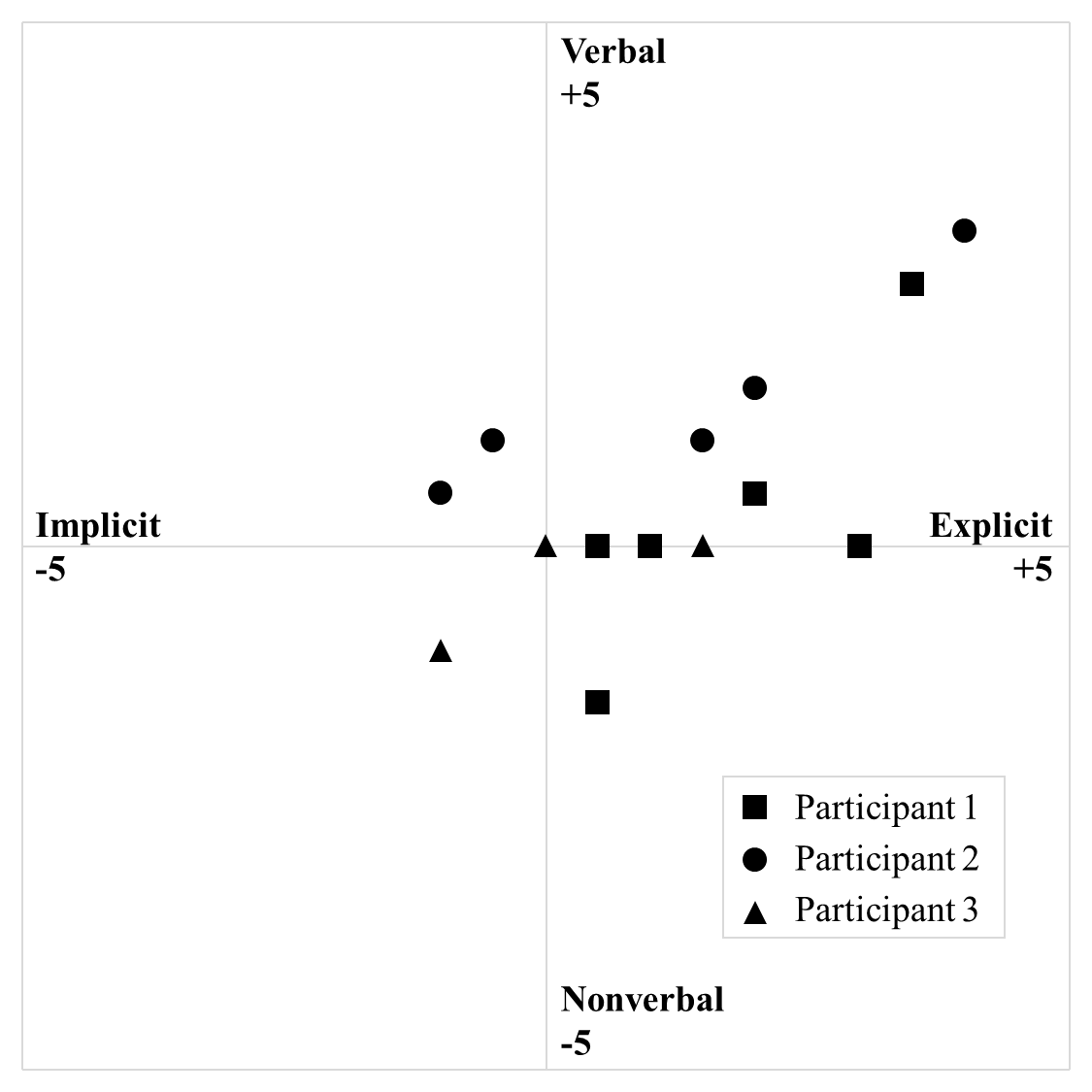

Supplement: Supplemental Material [file HJSR_A_1907526_SM8435.doc]
